# Melatonin supplementation improves N‐terminal pro‐B‐type natriuretic peptide levels and quality of life in patients with heart failure with reduced ejection fraction: Results from MeHR trial, a randomized clinical trial

**DOI:** 10.1002/clc.23796

**Published:** 2022-02-16

**Authors:** Shervin G. Hoseini, Kiyan Heshmat‐Ghahdarijani, Saeid Khosrawi, Mohammad Garakyaraghi, Davood Shafie, Marjan Mansourian, Hamidreza Roohafza, Elham Azizi, Masoumeh Sadeghi

**Affiliations:** ^1^ Isfahan Cardiovascular Research Center, Cardiovascular Research Institute Isfahan University of Medical Sciences Isfahan Iran; ^2^ Department of Physical Medicine and Rehabilitation, School of Medicine Isfahan University of Medical Sciences Isfahan Iran; ^3^ Heart Failure Research Center, Cardiovascular Research Institute Isfahan University of Medical Sciences Isfahan Iran; ^4^ Cardiac Rehabilitation Research Center, Cardiovascular Research Institute Isfahan University of Medical Sciences Isfahan Iran

**Keywords:** brain natriuretic peptide, heart failure, melatonin, quality of life

## Abstract

**Background:**

Melatonin, the major secretion of the pineal gland, has beneficial effects on the cardiovascular system and might advantage heart failure with reduced ejection fraction (HFrEF) by attenuating the effects of the renin–angiotensin–aldosterone and sympathetic system on the heart besides its antioxidant and anti‐inflammatory effects.

**Hypothesis:**

We hypothesized that oral melatonin might improve echocardiographic parameters, serum biomarkers, and a composite clinical outcome (including quality of life, hospitalization, and mortality) in patients with HFrEF.

**Methods:**

A placebo‐controlled double‐blinded randomized clinical trial was conducted on patients with stable HFrEF. The intervention was 10 mg melatonin or placebo tablets administered every night for 24 weeks. Echocardiography and measurements of N‐terminal pro‐B‐type natriuretic peptide (NT‐Pro BNP), high‐sensitivity C‐reactive protein, lipid profile, and psychological parameters were done at baseline and after 24 weeks.

**Results:**

Overall, 92 patients were recruited, and 85 completed the study (melatonin: 42, placebo: 43). Serum NT‐Pro BNP decreased significantly in the melatonin compared with the placebo group (estimated marginal means for difference [95% confidence interval]: 111.0 [6.2–215.7], *p* = .044). Moreover, the melatonin group had a significantly better clinical outcome (0.93 [0.18–1.69], *p* = .017), quality of life (5.8 [0.9–12.5], *p* = .037), and New York Heart Association class (odds ratio: 12.9 [1.6–102.4]; *p* = .015) at the end of the trial. Other studied outcomes were not significantly different between groups.

**Conclusions:**

Oral melatonin decreased NT‐Pro BNP and improved the quality of life in patients with HFrEF. Thus it might be a beneficial supplement in HFrEF.

## 1 INTRODUCTION

Heart failure with reduced ejection fraction (HFrEF) is a worldwide growing problem. Several effective medications have been developed which reduce symptoms and increase the survival of these patients.^1^ However, the disease process is frequently ongoing. Underlying intrinsic mechanisms such as mitochondrial abnormalities and cardiomyocyte hypertrophy and fibrosis and extrinsic mechanisms like overactivation of the sympathetic nervous system (SNS) and renin–angiotensin–aldosterone system (RAAS) promote cardiomyocyte apoptosis and gradual deterioration of the left ventricular function.^2^ This process is not entirely preventable by current heart failure (HF) treatments^2^; thus, alternative medications targeting different mechanisms or having synergistic effects with existing drugs might improve the patients' overall health and quality of life.

Melatonin is mainly secreted by the pineal gland with the primary role of coordinating the circadian rhythm. Recently, it has been shown that melatonin has beneficial effects on the cardiovascular system by its cytoprotective and antioxidant properties and by ameliorating mitochondrial dysfunction and regulating the endocrine system, such as SNS and RAAS.^3^, ^4^, ^5^, ^6^ Regarding the field of HF, numerous studies demonstrated that melatonin supplementation prevents the development or progression of the disease in animal models of both ischemic and nonischemic HF.^5^ Also, observational studies in humans have shown lower melatonin levels in patients with severe HF or those with hypertensive cardiomyopathy who developed HF.^7^, ^8^ However, the effect of exogenous melatonin on patients with established HFrEF is unclear, and it has rarely been assessed in clinical trials.^9^ Thus, we aimed to evaluate the effect of oral melatonin supplementation on echocardiographic parameters, serum N‐terminal pro‐B‐type natriuretic peptide (NT‐Pro BNP), and clinical outcomes in patients with HFrEF.

## 2 MATERIALS AND METHODS

### 2.1 Study design

This report adheres to the Consolidated Standards of Reporting Trials (CONSORT).^10^ The research was designed and conducted according to the general principles outlined in the Declaration of Helsinki. The Ethical Committee of Isfahan University of Medical Sciences had approved the study (IR.MUI.MED.REC.1397.067). Written informed consent was obtained from all participants. The trial's protocol was registered in ClinicalTrials.gov (NCT03894683).

The MeHR trial was a double‐blinded randomized placebo‐controlled clinical trial with two parallel arms, conducted from January 2019 to August 2020 in outpatient Chamran Cardiology Clinics. The design and methods of the study are fully described elsewhere.^11^

### 2.2 Study participants

Participants were selected from patients with a definite diagnosis of HFrEF (left ventricular ejection fraction [LVEF] < 40%) who were symptomatic (New York Heart Association [NYHA] class II or III) and regularly attended a specialist in mentioned clinics. In addition, they had to be clinically stable for at least 3 months and on essential drugs for HFrEF according to the 2016 ESC guidelines, that is, receiving maximum tolerated doses of an angiotensin‐converting enzyme inhibitor/angiotensin‐receptor blocker, a beta‐blocker, and spironolactone/eplerenone unless contraindicated, and diuretics if needed. The medications and other indicated treatments were prescribed by the specialists who referred the patients for participating in the study. Patients suspected to need device therapy (pacemaker or implantable cardioverter‐defibrillator) within the next 6 months were not enrolled.

Details of eligibility criteria have already been declared.^11^ Eligible patients were enrolled and randomized by block randomization to the placebo or melatonin groups. The participants, primary investigator, and all outcome assessors were blinded to the study groups.

### 2.3 Interventions

The intervention was 10 mg melatonin or placebo tablets, identical in shape and physical properties made by Sepid Teb (Sepid Teb Co.) and prescribed for at least 24 weeks, one tablet at bedtime. The drug was delivered to the patients in identical boxes containing 100 tablets and they were requested to return the unconsumed pills in the scheduled follow‐ups. Patients' adherence and the adverse effects were assessed regularly by telephone calls, and patients attended the study center after 12 and 24 weeks of intervention for outcome evaluation and pill count to objectively evaluate adherence to the intervention.

### 2.4 Outcomes

The primary outcomes of the study were variations in LVEF and left ventricular end‐diastolic diameter (LVEDD), measured by echocardiography at Week 24 relative to the baseline, and changes in serum levels of NT‐Pro BNP at Week 24. Also, a compound clinical score was calculated at Week 24 for each patient, composed from all‐cause mortality, any hospitalization for HF exacerbation, and changes in quality of life measured by the Minnesota Living with Heart Failure Questionnaire (MLHFQ).^12^ Each component was scored according to Table S1 and the sum of scores was calculated as the compound clinical outcome.

Clinical events were collected at baseline and during the study. Hospitalization was recorded from patients' medical documents, and mortality was recognized by verbal autopsy. An expert blinded committee of three cardiologists confirmed the events by evaluating the patient's medical records.

Other outcomes analyzed in this report were serum levels of high‐sensitivity C‐reactive protein (hs‐CRP), lipid profile, and liver and renal function tests measured at baseline and end of the intervention (Week 24), as well as results of questionnaires measuring sleep quality (Pittsburgh Sleep Quality Index), anxiety (the trait part of Spielberger Anxiety Inventory), and depression (Beck Depression Inventory‐II).

Transthoracic 2D‐color echocardiography was done by a single specialist, blinded to the study groups, via the Simpson biplane method with concomitant electrocardiography monitoring (GE Vivid 3.0; General Electric Vingmed Ultrasound). The intraobserver variability was determined by reassessing the stored images of 30 random baseline echocardiography measurements at least 1 month later. The intra‐class correlation coefficient of .97, .98, and .95 was obtained for LVEDD, left ventricular end‐systolic diameter (LVESD), and LVEF, respectively.

A 5 ml fasting blood sample was drawn from each participant at baseline and Week 24. Routine blood tests (lipid profile and liver and renal function tests) were performed by an autoanalyzer (Hitachi 902), and serum aliquots were stored at −80 for further analysis. Serum NT‐pro BNP was measured by a commercial enzyme‐linked immunosorbent assay (ELISA Kit) (Bioassay Technology Laboratory) according to the kit instruction. Serum hs‐CRP levels were determined by immunoturbidimetry (Pars Azmoon Inc).

Validated Persian versions of relevant questionnaires were used in this study. The MLHFQ is a widely used health‐related quality of life questionnaire for patients with HF with acceptable validity.^13^ It is a six‐point Likert scale questionnaire composed of 21 questions covering the physical and emotional domains, and the total score ranges from 0 to 105 (*from best* to *worst*).^13^ The Pittsburgh Sleep Quality Index is a valid tool for screening the sleep quality and quantity consisting of different subdomains and a total score of 0–21. Scores more than five indicate poor sleep quality.^14^ We used the trait part of Spielberger Anxiety Inventory (range: 20–80) and Beck Depression Inventory‐II (range: 0–63) to evaluate the psychological status of our patients before and after the intervention, both on a Four‐Likert scale.^15^, ^16^ All questionnaires were self‐administered; however, a trained questioner read the items and recorded the answers for illiterate patients.

### 2.5 Sample size and statistical methods

The sample size of 90 was calculated to find a 5% difference in LVEF between groups at the significance level of 0.05 (two‐tailed) and the power of 90%, based on a study by Garakyaraghi et al.^9^ Statistical analyses was performed by SPSS version 22 (IBM SPSS Statistics). The data are presented as median (interquartile range) or percentage when appropriate. The independent sample *T*‐test/Mann–Whitney *U* test or *χ* ^2^ test were used to compare baseline variables and the rate of adverse events between groups. The primary and secondary outcomes were analyzed using analysis of covariance, all adjusted for baseline values and other covariates as appropriate for the examined outcome. The missing data met the assumptions of missing completely at random. An intention‐to‐treat analysis approach was employed by including all cases with at least one measurement. A statistically significant level of less than 0.05 was acceptable for two‐sided tests.

## 3 RESULTS

### 3.1 Study flow and baseline characteristics

**Figure 1:**
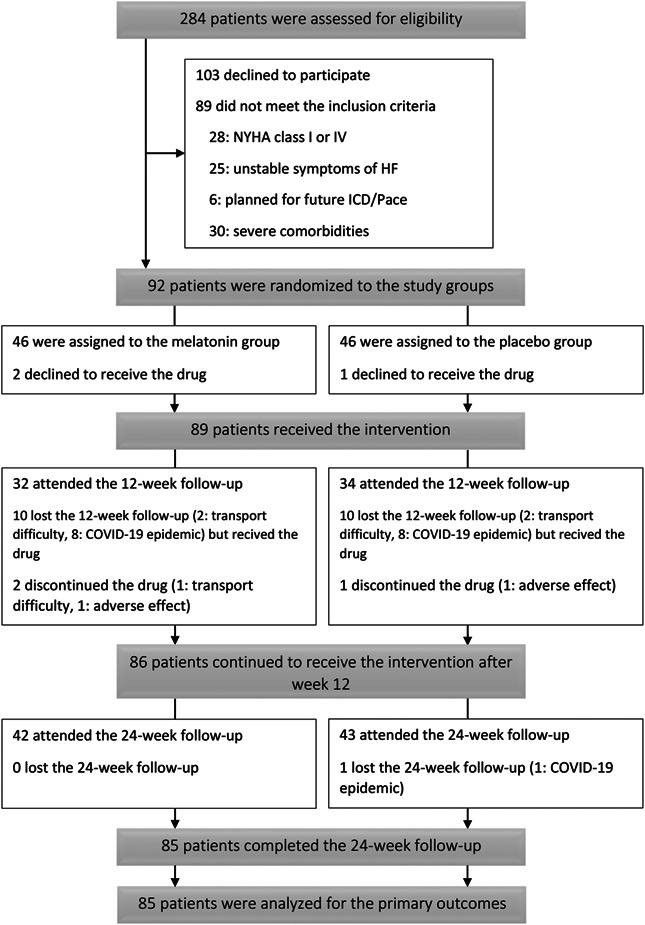
CONSORT flow diagram of MeHR trial

Figure 1 demonstrates the study flow diagram. We screened 284 patients with a definite diagnosis of HFrEF and finally enrolled 92 patients, 46 randomized to each group. The median age of the participants was 61.5 (30–82 years), with a male predominance of 87% and a median LVEF of 29% (13%–40%). Most of the patients had an ischemic etiology for their disease (87%) and were categorized in NYHA class II (76.1%). The patients' major baseline characteristics and study investigations were balanced between study groups, as are presented in Tables 1 and 2. Finally, 85 patients completed the last study follow‐up (Figure 1). The study team canceled the 12‐week follow‐up optionally for some patients due to the coronavirus disease 2019 (COVID‐19) epidemic in the region. Thus, we had 20 missing for the 12‐week follow‐up, mainly because of the COVID‐19 pandemic.

**Table 1:** Baseline demographic and clinical characteristics of the MeHR trial patients

Baseline characteristic	Control (*N* = 46)	Melatonin (*N* = 46)	*p* Value
Age, year	58.5 (54.0–67.2)	63.5 (56.7–70.2)	.123
Sex			1.000
Male	40 (87.0%)	40 (87.0%)	
Female	6 (13%)	6 (13%)	
Body mass index (kg/m^2^)	26.9 (25.0–28.1)	27.2 (24.2–28.9)	.541
Mean blood pressure (mmHg)	87.5 (77.4–93.2)	89.7 (82.3–100.2)	.049
Pulse rate (beat/min)	68.0 (58.5–77.7)	71.0 (64.0–79.0)	.382
Etiology of heart failure			.354
Ischemic	38 (82.6%)	42 (91.3%)	
Nonischemic	8 (17.4%)	4 (8.7%)	
Duration of the disease (year)	3.0 (1.0–7.0)	2.5 (1.1–5.0)	.943
NYHA class			1.000
Class II	35 (76.1%)	35 (76.1%)	
Class III	11 (23.9%)	11 (23.9%)	
Current medications			
Diuretic	26 (56.5%)	15 (32.6%)	.018
ACE inhibitor/ARB	32 (69.6%)	31 (67.4%)	1.000
Beta‐blocker	42 (91.3%)	34 (73.9%)	.052
Calcium‐blocker	3 (6.5%)	2 (4.3%)	1.000
MRA	20 (43.5%)	17 (37.0%)	.671
SGLT‐2 inhibitor	0 (0%)	0 (0%)	
ARNI	1 (2.2%)	0 (0%)	
Digoxin	5 (10.9%)	9 (19.6%)	.385
Nitrate	9 (19.6%)	15 (32.6%)	.235
Statin	37 (80.4%)	38 (82.6%)	1.000
Antiplatelet	39 (84.8%)	41 (89.1%)	.758
Pacemaker/ICD	9 (19.6%)	4 (8.7%)	.231
Atrial fibrillation	2 (4.3%)	0 (0.0%)	
History of MI	27 (58.7%)	33 (71.7%)	.274
History of CABG	16 (34.8%)	14 (30.4%)	.824
Diabetes mellitus	12 (26.1%)	16 (34.8%)	.497
Renal disease	1 (2.2%)	5 (10.9%)	.203
Pulmonary disease	5 (10.9%)	3 (6.5%)	.714
Ever smoker	24 (52.2%)	21 (45.7%)	.677
Alcohol use	1 (2.2%)	3 (6.5%)	.617
Opium use	12 (26.1%)	7 (15.2%)	.303

*Note*: Data are expressed as median (interquartile range) or number (%).

Abbreviations: ACE, angiotensin‐converting enzyme; ARB, angiotensin‐receptor blocker; ARNI, angiotensin receptor‐neprilysin inhibitor; CABG, coronary artery bypass graft; ICD, implantable cardioverter‐defibrillator; MI, myocardial infarction; MRA, mineralocorticoid receptor antagonists; NYHA, New York Heart Association; SGLT‐2, sodium‐glucose cotransporter‐2.

**Table 2:** Baseline investigations of the MeHR trial patients

Baseline investigation	Control (*N* = 46)	Melatonin (*N* = 46)	*p* Value
Echocardiographic parameters			
LVEF (%)	30.0 (20.7–35.0)	28.5 (21.0–35.2)	.857
LVEDD (cm)	5.5 (4.9–6.2)	5.6 (5.0–6.0)	.511
LVESD (cm)	4.4 (4.1–5.1)	4.6 (4.0–4.9)	.886
Serum markers			
NT‐Pro BNP (ng/L)	318 (281–375)	319 (280–374)	.985
hs‐CRP (mg/L)	0.90 (0.30–3.00)	1.20 (0.37–2.62)	.928
Triglyceride (mg/dl)	126 (86–162)	132 (108–190)	.215
Total cholesterol (mg/dl)	144 (132–181)	159 (138–190)	.303
LDL (mg/dl)	75 (63–99)	83 (70–104)	.263
HDL (mg/dl)	44 (40–49)	42 (36–50)	.380
Blood urea nitrogen (mg/dl)	16.0 (13.0–19.5)	15.0 (15.0–20.0)	.629
Creatinine (mg/dl)	1.2 (1.0–1.4)	1.2 (1.1–1.3)	.288
eGFR (ml/min/1.73 m^2^)	59.3 (50.0–70.3)	56.4 (51.2–63.7)	.077
Aspartate transaminase (U/L)	21.0 (16.7–25.0)	20.0 (16.0–25.0)	.551
Alanine aminotransferase (U/L)	20.5 (12.7–32.2)	22.0 (12.0–29.5)	.880
Psychological parameters			
Quality of life, MLHFQ score	25.0 (16.0–41.2)	23.0 (15.0–30.5)	.110
Sleep quality, PSQI score	7.0 (5.0–10.2)	6.0 (8.0–13.0)	.230
Anxiety, STAI score	44.0 (36.0–52.5)	42.0 (34.5–47.5)	.107
Depression, BDI‐II score	13.0 (8.0–24.0)	13.0 (9.0–17.7)	.325

*Note*: Data are expressed as median (interquartile range) or number (%).

Abbreviations: BDI‐II, Beck Depression Inventory‐II; eGFR, estimated glomerular filtration rate; HDL, high‐density lipoprotein cholesterol; hs‐CRP, high‐sensitivity C‐reactive protein; LDL, low‐density lipoprotein cholesterol; LVEDD, left ventricular end‐diastolic diameter; LVEF, left ventricular ejection fraction; LVESD, left ventricular end‐systolic diameter; MLHFQ, Minnesota Living with Heart Failure Questionnaire; NT‐Pro BNP, N‐terminal pro B‐type natriuretic peptide; NYHA, New York Heart Association; PSQI, Pittsburgh Sleep Quality Index; STAI, Spielberger Trait Anxiety Inventory.

Pill count showed a high adherence to the intervention in participants who completed the study, ranging from 80% to 100% (97.8% in the melatonin and 98.7% in the placebo group).

### 3.2 Echocardiographic parameters and serum markers of HF

The echocardiographic parameters of LVEF, LVEDD, and LVESD were not affected by the intervention after adjustment for the age and HF type (ischemic vs. nonischemic). The estimated marginal means (EMMs) of serum NT‐Pro BNP at Week 24 was significantly lower in the melatonin group relative to the placebo group after adjustment for predefined parameters of age, sex, body mass index, and estimated glomerular filtration rate (EMM [95% confidence interval {CI}] for the difference between groups: 111.0 [6.2–215.7]; *p* = .044). Serum levels of hs‐CRP were not different between groups at week 24 (EMM [95% CI] for difference: 0.41 [−1.49 to 2.31]; *p* = .677; adjusted for age and sex). Table 3 shows the EMM for primary and secondary outcomes of the trial.

**Table 3:** Estimated marginal means for outcomes of the MeHR trial

Outcome	Control (95% CI) (*N* = 43)	Melatonin (95% CI) (*N* = 42)	*p* Value
Echocardiographic parameters			
LVEF (%)	29.1 (27.3–29.5)	29.5 (27.1–31.8)	.816
LVEDD (cm)	5.2 (5.0–5.5)	5.1 (4.7–5.4)	.442
LVESD (cm)	4.3 (4.1–4.6)	4.2 (3.9–4.6)	.672
Serum markers of HF			
NT‐Pro BNP (ng/L)	332.1 (253.5–410.7)	221.1 (148.9–293.2)	.044
hs‐CRP (mg/L)	2.01 (0.67–3.34)	1.60 (0.18–3.01)	.677
Composite clinical outcome^a^ (score)	0.02 (−0.49 to 0.54)	0.96 (0.39–1.52)	.017
Blood tests			
Triglyceride (mg/dl)	147.8 (131.0–164.6)	136.2 (119.8–152.6)	.331
Total cholesterol (mg/dl)	162.6 (152.1–173.0)	155.4 (145.2–165.7)	.332
LDL (mg/dl)	84.1 (76.2–92.0)	81.2 (73.6–88.9)	.598
HDL (mg/dl)	46.8 (44.0–49.7)	46.2 (43.5–49.0)	.761
Blood urea nitrogen (mg/dl)	16.6 (14.7–18.4)	16.6 (14.6–18.6)	.992
Creatinine (mg/dl)	1.26 (1.17–1.36)	1.25 (1.16–1.35)	.899
Aspartate transaminase (U/L)	22.5 (19.7–25.2)	20.0 (17.0–23.0)	.228
Alanine aminotransferase (U/L)	23.0 (18.7–27.3)	18.6 (13.9–23.4)	.179
Psychological parameters			
Quality of life, MLHFQ score	28.0 (23.2–32.8)	22.2 (17.4–27.0)	.037
Sleep quality, PSQI score	5.9 (4.6–7.3)	5.3 (3.8–6.9)	.544
Anxiety, STAI score	38.7 (35.1–42.3)	39.7 (35.9–43.6)	.690
Depression, BDI‐II score	13.8 (10.9–16.6)	13.4 (10.0–16.8)	.877

Abbreviations: BDI‐II, Beck Depression Inventory‐II; HDL, high‐density lipoprotein cholesterol; hs‐CRP, high‐sensitivity C‐reactive protein; LDL, low‐density lipoprotein cholesterol; LVEDD, left ventricular end‐diastolic diameter; LVEF, left ventricular ejection fraction; LVESD, left ventricular end‐systolic diameter; MLHFQ, Minnesota Living with Heart Failure Questionnaire; NT‐Pro BNP, N‐terminal pro B‐type natriuretic peptide; PSQI, Pittsburgh Sleep Quality Index; STAI, Spielberger Trait Anxiety Inventory.

^a^ Composite clinical outcome consisted of all‐cause mortality, hospitalization for HF exacerbation, and changes in quality of life measured by MLHFQ.

### 3.3 The compound clinical outcome

During the study, 12 hospitalizations occurred among patients who completed the study, of which 3 were because of HF exacerbation (one from five in melatonin and two from six in the placebo group). Quality of life measured by MLHFQ improved at least five scores in 39 patients (22 [52.4%] in the melatonin and 17 [39.5%] in the placebo group) and worsened at least five scores in 13 persons (6 [14.3%] in the melatonin and 7 [16.3%] in the placebo group) after 24 weeks. The composite clinical outcome was significantly better in the melatonin than in the placebo group (EMM [95% CI] for difference: 0.93 [0.18–1.69]; *p* = .017).

The MLHFQ was measured at Week 12, too; the generalized estimating equation model with three time‐points as within‐subject variable and age and sex as covariates showed a significant effect for treatment (*p* = .019) and treatment and time interaction (*p* < .001) (EMM [95% CI] for difference: 5.8 [0.9–12.5]) in favor of the melatonin group. The NYHA class of the patients measured before and after 24 weeks of intervention did not change in 75 patients, improved in 5 patients (four in the melatonin group), and worsened in 5 patients (four in the control group). All patients were categorized in NYHA classes II and III after the intervention. The logistic regression adjusted for baseline values of NYHA class, age, and sex showed a significant effect for intervention on the NYHA class of the patients in the melatonin group (odds ratio [95% CI]: 12.9 [1.6–102.4]; *p* = .015).

### 3.4 Psychological parameters

In our study, 24 weeks of melatonin supplementation did not affect the score of sleep quality, anxiety, or depression questionnaires (Table 3). Moreover, none of the subdomain scores of the sleep questionnaires (sleep duration, latency, efficiency, disturbances, medications, daytime dysfunction, and subjective sleep quality) were significantly different before and after treatment between groups.

### 3.5 Lipid profile and liver and renal function tests

Although the mean differences of lipid profiles from baseline to Week 24 were substantially in favor of the melatonin group (mean difference [mg/dl] [95% CI]: 13.1 [−4.6 to 30.9] for triglyceride; 9.3 [−2.6 to 21.3] for total cholesterol; 5.5 [−3.5 to 14.6] for low‐density lipoprotein [LDL]), however, they did not reach statistically significant difference. In addition, the renal function tests were not significantly affected by the treatment during the study. The aspartate transaminase and alanine aminotransferase levels were somewhat improved in the melatonin group, but they did not reach a statistically significant difference (Table 3).

### 3.6 Drug‐related adverse effects

From 92 allocations, three did not receive the intervention (two in the melatonin group and one in the placebo group). Throughout the study, 13 of 89 patients reported at least one type of drug‐related adverse effect (Table 4). However, the rate of adverse effects was not statistically different between the groups (9 [20.5%] in the melatonin group and 4 [8.9%] in the control group; *p* = .144). One patient in each group left the study because of skin eruptions after consumption of the drug. No serious adverse effect was seen during the study.

**Table 4:** Adverse events during the MeHR trial

Adverse events	Control (*N* = 45)	Melatonin (*N* = 44)
Hospitalization	6 (14.0%)	5 (11.9%)
Adverse drug effects^a^	4 (8.9%)	9 (20.5%)
Morning sleepiness		1
Insomnia	3	
Nightmare	2	1
Anxiety		1
Skin eruptions	1	1
Dry mouth		1
Headache		1
lassitude		1
Dyspepsia		1
Hypotension		1
Decreased appetite		1

^a^ Some patients had more than one complaint.

## 4 DISCUSSION

This study showed that oral melatonin supplementation lowered the serum levels of NT‐Pro BNP in patients with HFrEF and improved their disease‐specific quality of life. Moreover, patients who received melatonin had a better composite clinical outcome, and their overall NYHA class improved after 24 weeks of intervention. However, melatonin did not affect the echocardiographic parameters of the patients.

In a study, Garakyaraghi et al.^9^ showed that 3 mg oral melatonin for 2 months significantly improved LVEF and NYHA class in patients with HFrEF. We detected an improvement in NYHA class of the melatonin group, too; however, we found no significant change in echocardiographic parameters in any groups. This discrepancy might be due to differences in characteristics of the study participants, their current medications, and the etiology of the disease. As our patients had a mean disease duration of 3 years and generally received optimum therapy, changes in LVEF and other parameters by transthoracic echocardiography might be unlikely because of residual chronic irreversible myocardial remodeling and fibrosis. On the other hand, using global longitudinal strain technology, a more sensitive method to evaluate LV systolic function, could detect subtle changes in myocardial performance, although it was not performed in our study because of limitations.^17^

Melatonin decreased serum NT‐Pro BNP compared to placebo, but it did not affect hs‐CRP as an inflammatory marker. The positive effect of melatonin on NT‐Pro BNP level might be due to the synergistic effect of melatonin with angiotensin‐converting enzyme inhibitor/angiotensin‐receptor blocker and beta‐blockers in the modulation of RAAS and SNS.^3^ In vitro and in vivo studies suggest that melatonin antagonizes the adverse effects of angiotensin on the cardiovascular system by its antioxidant, anti‐inflammatory, and antihypertensive effects.^3^ However, the direct effect of melatonin on inhibition of the RAAS system is controversial in different studies.^18^, ^19^ Furthermore, evidence proposes that exogenous melatonin protects the myocardium from excess epinephrine toxicity and reduces adrenergic activity in humans.^20^, ^21^

Melatonin's ability to improve mitochondrial dysfunction might alleviate myocardial function and decrease overload. In experimental studies, melatonin administration inhibited the opening of mitochondrial permeability transition pores in myocytes of the aging ischemic hearts and reversed the functional and biochemical changes in ischemic HF models.^4^, ^5^ In addition, melatonin prevented myocyte apoptosis and autophagy dysfunction by Atg5‐ and Akt/mTOR‐dependent pathways in pressure overload‐induced cardiac hypertrophy.^22^ Also, it protected against diabetic cardiomyopathy via the key melatonin receptor, retinoid‐related orphan receptor α, through various downstream signaling pathways.^6^

The beneficial effect of melatonin was also detectable on the composite clinical outcome, including mortality, hospitalization due to HF decompensation, and quality of life. However, we had no mortality and few hospitalizations during the study. Indeed, most of the clinical benefit of melatonin was its effect on the quality of life.

Melatonin did not affect sleep quality and quantity, anxiety, and depression levels during this investigation. There are discrepancies among previous studies and recent systematic reviews regarding the effect of melatonin on sleep patterns; overall, it seems that the melatonin effect is dependent on several factors such as dose, duration of use, pharmacokinetic of the melatonin formulations, and the background characteristics of the studied population.^23^, ^24^

Furthermore, the effect of melatonin on mental health is inconsistent. Jafari‐Vayghan et al.^25^ found that 20 mg melatonin for 8 weeks improved the overall quality of life and physical dimension of MLHFQ scores in cachectic patients with HF but had no effect on its emotional dimension. Melatonin ameliorated depressive‐like behaviors in animal models^26^; however, the evidence for its role in human mood disorders is not conclusive.^27^ Extensive trials specifically designed for these purposes would be helpful to the field of cardiac diseases.

Melatonin is supposed to improve serum lipid profile in various target populations, possibly by directly regulating lipid metabolism and reducing the detrimental effect of oxidizing lipoproteins on the cardiovascular system.^28^ Our insignificant results despite substantial improvement of total cholesterol, triglyceride, and LDL might be due to low sample size or lower levels of baseline lipids in our patients, as Mohammadi‐Sartang et al.^29^ demonstrated that higher doses of melatonin (>8 mg) and lower baseline total cholesterol (<200 mg/dl) are associated with a significant decline in serum cholesterol.^29^

We performed liver and renal function tests for the primary purpose of detecting any drug‐related adverse effects in our patients. On the other hand, several studies have shown melatonin to ameliorate nonalcoholic fatty liver disease and decrease liver transaminases in concordance with moderately lower levels of these enzymes in our melatonin group.^30^ Statins are valuable drugs in HFrEF irrespective of HF etiology, and increased liver transaminases are a concern for their prescription; thus, future studies can focus on the probable positive effect of melatonin on tolerability to the statins in these patients.^31^ Overall, drug‐related adverse effect profiles of our patients indicated that long‐term melatonin supplementation with this dose is safe for patients with HFrEF.

We found an overall 35% reduction in NT‐Pro BNP concentration in melatonin compared with the placebo group relative to baseline values; while some clinical trials defined a reduction of 30% in NT‐Pro BNP level during treatment as a primary endpoint,^32^, ^33^ the clinical impact of this finding is not clear. Several studies have shown that serum levels of natriuretic peptides can accurately predict morbidity and mortality in patients with chronic HF and their treatment‐related changes well correlate with HF hospitalization.^34^, ^35^ However, the magnitude of difference between treatment and control groups in clinical trials leading to significant clinical benefits is not defined precisely.^35^ Recent studies recommend multiple biomarkers such as serum troponin in addition to NT‐Pro BNP to determine the long‐term effect of interventions on HF prognosis, which might be a limitation to our study.^34^

The low sample size was also a limitation of the study, which might have caused some insignificant results. This low sample size might also have affected the clinical outcomes such as death and hospitalization despite a respectively long follow‐up and made any conclusion about these outcomes unreliable. However, this sample size seems enough to evaluate the primary outcomes. Also, the high number of missing in the first follow‐up was another limitation imposed by the urgent COVID‐19 epidemics, although none of the primary outcomes had been planned to be evaluated at the first follow‐up.

Extended follow‐up time relative to other clinical trials using melatonin in cardiovascular diseases is a strength of the MeHR trial. Also, up to our knowledge, this is the first study that includes biological biomarkers, structural parameters, and patient‐oriented outcomes to evaluate the melatonin effect in HFrEF.

Overall, melatonin might lower serum NT‐Pro BNP and improve disease‐specific health‐related quality of life in patients with HFrEF. Thus it could be a valuable supplement for these patients. Further studies in subgroups of patients with HF, such as diabetic or hypertensive cardiomyopathic patients, and sensitive evaluation methods for cardiac function might provide new information in this regard.

## CONFLICT OF INTERESTS

The authors declare that there are no conflict of interests.

## Supporting information

Supporting information.

## Data Availability

The data that support the findings of this study are available from the corresponding author upon reasonable request.

## References

[1] Doimo S , Pavan D . Novelties in therapy of chronic heart failure. Heart Fail Clin. 2021;17:255‐262. 33673949 10.1016/j.hfc.2021.01.006

[2] Sabbah HN . Silent disease progression in clinically stable heart failure. Eur J Heart Fail. 2017;19:469‐478. 27976514 10.1002/ejhf.705 PMC5396296

[3] Jafari‐Vayghan H . The effects of melatonin on neurohormonal regulation in cardiac cachexia: a mechanistic review. J Cell Biochem. 2019;120:16340‐16351. 31168891 10.1002/jcb.29151

[4] Baburina Y , Lomovsky A , Krestinina O . Melatonin as a potential multitherapeutic agent. J Pers Med. 2021;11:274. 33917344 10.3390/jpm11040274 PMC8067360

[5] Nduhirabandi F , Maarman GJ . Melatonin in heart failure: a promising therapeutic strategy? Molecules. 2018;23:1819. 10.3390/molecules23071819 PMC6099639 30037127

[6] Song YJ , Zhong CB , Wu W . Cardioprotective effects of melatonin: focusing on its roles against diabetic cardiomyopathy. Biomed Pharmacother. 2020;128:110260. 32447213 10.1016/j.biopha.2020.110260

[7] Dominguez‐Rodriguez A , Abreu‐Gonzalez P , Reiter RJ . The potential usefulness of serum melatonin level to predict heart failure in patients with hypertensive cardiomyopathy. Int J Cardiol. 2014;174:415‐417. 24768380 10.1016/j.ijcard.2014.04.044

[8] Girotti L , Lago M , Ianovsky O , et al. Low urinary 6‐sulfatoxymelatonin levels in patients with severe congestive heart failure. Endocrine. 2003;22:245‐248. 14709797 10.1385/ENDO:22:3:245

[9] Garakyaraghi M , Siavash M , Alizadeh MK . Effects of melatonin on left ventricular ejection fraction and functional class of patients with heart failure: a randomized, double‐blind, placebo‐controlled trial. J Res Med Sci. 2012;17:S13‐S16.

[10] Schulz KF , Altman DG , Moher D . ONSORT Statement: updated guidelines for reporting parallel group randomized trials. BMC Med. 2010;340:c332. PMC3116666 21686296

[11] Sadeghi M . Effect of melatonin on heart failure: design for a double‐blinded randomized clinical trial. ESC Heart Fail. 2020;7:3142‐3150. 32618134 10.1002/ehf2.12829 PMC7524054

[12] Taylor AL , Ziesche S , Yancy C , et al. Combination of isosorbide dinitrate and hydralazine in blacks with heart failure. N Engl J Med. 2004;351:2049‐2057. 15533851 10.1056/NEJMoa042934

[13] Garin O , Herdman M , Vilagut G , et al. Assessing health‐related quality of life in patients with heart failure: a systematic, standardized comparison of available measures. Heart Fail Rev. 2014;19:359‐367. 23681849 10.1007/s10741-013-9394-7

[14] Mollayeva T , Thurairajah P , Burton K , Mollayeva S , Shapiro CM , Colantonio A . The Pittsburgh sleep quality index as a screening tool for sleep dysfunction in clinical and non‐clinical samples: a systematic review and meta‐analysis. Sleep Med Rev. 2016;25:52‐73. 26163057 10.1016/j.smrv.2015.01.009

[15] Ghassemzadeh H , Mojtabai R , Karamghadiri N , Ebrahimkhani N . Psychometric properties of a Persian‐language version of the Beck Depression Inventory—second edition: BDI‐II‐PERSIAN. Depress Anxiety. 2005;21:185‐192. 16075452 10.1002/da.20070

[16] Kvaal K , Ulstein I , Nordhus IH , Engedal K . The Spielberger State‐Trait Anxiety Inventory (STAI): the state scale in detecting mental disorders in geriatric patients. Int J Geriatr Psychiatry. 2005;20:629‐634. 16021666 10.1002/gps.1330

[17] Tanaka H . Utility of strain imaging in conjunction with heart failure stage classification for heart failure patient management. J Echocardiogr. 2019;17:17‐24. 30443873 10.1007/s12574-018-0408-2

[18] Ohashi N , Ishigaki S , Isobe S . The pivotal role of melatonin in ameliorating chronic kidney disease by suppression of the renin‐angiotensin system in the kidney. Hypertens Res. 2019;42:761‐768. 30610209 10.1038/s41440-018-0186-2

[19] Simko F , Baka T , Krajcirovicova K , et al. Effect of melatonin on the renin‐angiotensin‐aldosterone system in l‐NAME‐induced hypertension. Molecules. 2018;23. 10.3390/molecules23020265 PMC6017142 29382124

[20] Vazan R , Ravingerova T . Protective effect of melatonin against myocardial injury induced by epinephrine. J Physiol Biochem. 2015;71:43‐49. 25572339 10.1007/s13105-014-0377-5

[21] Cagnacci A , Zanni AL , Veneri MG , Menozzi R , Volpe A , Rio GD . Influence of exogenous melatonin on catecholamine levels in postmenopausal women prior and during oestradiol replacement. Clin Endocrinol. 2000;53:367‐372. 10.1046/j.1365-2265.2000.01099.x 10971455

[22] Xu CN , Kong LH , Ding P , et al. Melatonin ameliorates pressure overload‐induced cardiac hypertrophy by attenuating Atg5‐dependent autophagy and activating the Akt/mTOR pathway. Biochim Biophys Acta, Mol Basis Dis. 2020;1866:165848. 32473999 10.1016/j.bbadis.2020.165848

[23] Gholami F , Moradi S , Rasae N , et al. Effect of melatonin supplementation on sleep quality: a systematic review and meta‐analysis of randomized controlled trials. J Neurol. 2021. 10.1007/s00415-020-10381-w 33417003

[24] Moroni I , Garcia‐Bennett A , Chapman J , Grunstein RR , Gordon CJ , Comas M . Pharmacokinetics of exogenous melatonin in relation to formulation, and effects on sleep: a systematic review. Sleep Med Rev. 2021;57:101431. 33549911 10.1016/j.smrv.2021.101431

[25] Jafari‐Vayghan H , Moludi J , Saleh‐Ghadimi S , Enamzadeh E , Seyed‐Mohammadzad MH , Alizadeh M . Impact of melatonin and branched‐chain amino acids cosupplementation on quality of life, fatigue, and nutritional status in cachectic heart failure patients: a randomized controlled trial. Am J Lifestyle Med. 2019;2006:1‐11. 10.1177/1559827619874044 PMC8848111 35185435

[26] Tonon AC , Pilz LK , Markus RP , Hidalgo MP , Elisabetsky E . Melatonin and depression: a translational perspective from animal models to clinical studies. Front Psychiatry. 2021;12:638981. 33897495 10.3389/fpsyt.2021.638981 PMC8060443

[27] De Crescenzo F , Lennox A , Gibson JC , et al. Melatonin as a treatment for mood disorders: a systematic review. Acta Psychiatr Scand. 2017;136:549‐558. 28612993 10.1111/acps.12755

[28] Loloei S , Sepidarkish M , Heydarian A , et al. The effect of melatonin supplementation on lipid profile and anthropometric indices: a systematic review and meta‐analysis of clinical trials. Diabetes Metab Syndr. 2019;13:1901‐1910. 31235113 10.1016/j.dsx.2019.04.043

[29] Mohammadi‐Sartang M , Ghorbani M , Mazloom Z . Effects of melatonin supplementation on blood lipid concentrations: a systematic review and meta‐analysis of randomized controlled trials. Clin Nutr. 2018;37:1943‐1954. 29191493 10.1016/j.clnu.2017.11.003

[30] Akhavan Rezayat A , Ghasemi Nour M , Bondarsahebi Y , et al. The effects of melatonin therapy on the treatment of patients with non‐alcoholic steatohepatitis: a systematic review and meta‐analysis on clinical trial studies. Eur J Pharmacol. 2021;905:174154. 34058202 10.1016/j.ejphar.2021.174154

[31] Bielecka‐Dabrowa A , Bytyci I , Von Haehling S , et al. Association of statin use and clinical outcomes in heart failure patients: a systematic review and meta‐analysis. Lipids Health Dis. 2019;18:188. 31672151 10.1186/s12944-019-1135-z PMC6822388

[32] Li X , Zhang J , Huang J , et al. A multicenter, randomized, double‐blind, parallel‐group, placebo‐controlled study of the effects of qili qiangxin capsules in patients with chronic heart failure. J Am Coll Cardiol. 2013;62(12):1065‐1072. 23747768 10.1016/j.jacc.2013.05.035

[33] Filippatos G , Anker SD , Böhm M , et al. A randomized controlled study of finerenone vs. eplerenone in patients with worsening chronic heart failure and diabetes mellitus and/or chronic kidney disease. Eur Heart J. 2016;37(27):2105‐2114. 27130705 10.1093/eurheartj/ehw132 PMC4946749

[34] Chow SL , Maisel AS , Anand I , et al. Role of biomarkers for the prevention, assessment, and management of heart failure: a scientific statement from the American Heart Association. Circulation. 2017;135:e1054‐e1091. 28446515 10.1161/CIR.0000000000000490

[35] Vaduganathan M , Claggett B , Packer M , et al. Natriuretic peptides as biomarkers of treatment response in clinical trials of heart failure. JACC Heart Fail. 2018;6(7):564‐569. 29501807 10.1016/j.jchf.2018.02.007

