# Supplementary material for: Melatonin supplementation improves N‐terminal pro‐B‐type natriuretic peptide levels and quality of life in patients with heart failure with reduced ejection fraction: Results from MeHR trial, a randomized clinical trial
Source: Clin Cardiol. 2022 Feb 16;45(4):417–26. doi: 10.1002/clc.23796 (PMC9019884; doi:10.1002/clc.23796)
Supplement: Supplementary file 1 — Supporting information. [file CLC-45-417-s001.docx]

Table 1. Composite clinical outcome score calculation in the study.

| **Components** | **Outcome** | **Score** |
| --- | --- | --- |
| Survival during the study | Survive | 0 |
|  | death | -3 |
| Hospitalization for heart failure during the study | No hospitalization | 0 |
|  | Any hospitalization | -1 |
| Change in MLHFQ scores before and after the intervention | Increase by ≥10 points | +2 |
|  | Increase by 5–9 points | +1 |
|  | Change by <5 points | 0 |
|  | Decrease by 5–9 points | -1 |
|  | Decrease by ≥10 points | -2 |
| **Total score** |  | **-6 to +2** |

Adopted from Taylor AL et al.^12^

MHLFQ: Minnesota Living with Heart Failure Questionnaire.
